# Financially supported roles for pharmacists in international settings

**DOI:** 10.1093/haschl/qxag277

**Published:** 2026-10-10

**Authors:** Melody Ryan, Michaela Almgren, Ian Bates, Clark D Kebodeaux, T Joseph Mattingly, Kathryn A Morbitzer, Christopher K Surratt

**Affiliations:** Department of Pharmacy Practice & Science, University of Kentucky College of Pharmacy, Lexington, KY 40536, United States; Department of Clinical Pharmacy and Outcome Science, University of South Carolina College of Pharmacy, Columbia, SC 29208, United States; University College London, UCL School of Pharmacy, 29-39 Brunswick Square, London WC1N 1AX, UK; Department of Pharmacy Practice & Science, University of Kentucky College of Pharmacy, Lexington, KY 40536, United States; Department of Pharmacotherapy, University of Utah College of Pharmacy, Salt Lake City, UT 84112, United States; Division of Practice Advancement and Clinical Education, University of North Carolina Eshelman School of Pharmacy, 301 Pharmacy Lane, CB 7355 Chapel Hill, NC 27599, United States; James L. Winkle College of Pharmacy, University of Cincinnati, Ohio 45267, United States

**Keywords:** pharmacist services, healthcare system models, health system financing, international, pharmacist reimbursement, comparative health policy, healthcare workforce, global health systems, financial sustainability

## Abstract

A May 2025 workshop convened by the National Academies of Sciences, Engineering, and Medicine examined the sustainability of the pharmacy profession amid pharmacy closures, workforce strain, and misaligned reimbursement in the United States. Its discussion of financially supported pharmacist roles focused on US models and identified a gap in attention to international payment structures. This article examines how pharmacists are paid across international health systems, using a framework of who pays for care, who owns delivery infrastructure, who provides services, and who decides coverage and reimbursement. Cases from the United Kingdom, Germany, Canada, South Korea, Nigeria, and Singapore illustrate how financing and governance arrangements shape pharmacist roles. Across the cases, reimbursement embedded in system financing, standardized service definitions, and alignment between scope of practice and payment were common features where pharmacist services had been incorporated into routine care. The analysis identifies considerations for advancing financially viable pharmacist services in the United States.

## Teaser

Pharmacist services are increasingly recognized as essential to health system performance, yet sustainable payment mechanisms remain underdeveloped in the United States. This article compares how health systems in the United Kingdom, Germany, Canada, South Korea, Nigeria, and Singapore finance pharmacist services and finds that these services are more readily integrated into routine care when reimbursement, scope of practice, and regulatory authority are aligned. The cases offer policy insight for addressing pharmacy closures and workforce strain in the United States.

Key Points

- Pharmacist services appear most durable when payment is structurally embedded. In the countries examined, roles established through legislation or regulated insurance frameworks appeared more stable than those dependent on pilots or discretionary funding, though this descriptive comparison cannot establish cause.
- US reform will require a hybrid approach. No single international model offers a template for the United States; workable solutions will need to combine public purchasing, insurance-based reimbursement, and regulated private delivery across federal and state levels.

## Introduction

A May 2025 workshop of the National Academies of Sciences, Engineering, and Medicine (NASEM) convened leaders in pharmacy education and practice to discuss the sustainability of the profession in the context of widespread US pharmacy closures, reimbursement inequities, rising medication costs, and drug shortages.^1^ The workshop focused on four pillars: pharmacy deserts, well-being, financial sustainability, and next-generation training and education with participants concluding with a road map of transformative strategies.^1^

This paper examines how pharmacists are financially supported across international settings. Prior literature has described pharmacist remuneration; this analysis organizes international examples around four foundational questions, who pays, who owns, who provides, and who decides, to explain how financing and regulatory alignment enable durable pharmacist roles. The purpose is not to identify an ideal health system or recommend adopting another country's model, but to identify recurring mechanisms that facilitate or constrain financially supported pharmacist services.

The country cases were chosen to show variation across the four health system models, not to provide comprehensive regional coverage or direct transferability to the United States. England, within the United Kingdom's Beveridge system, provides the primary case because of its nationally commissioned pharmacy services. Germany is the prototype of the Bismarck model and illustrates negotiated statutory insurance reimbursement. Canada and South Korea illustrate national health insurance with mixed public-private delivery. Nigeria and Singapore illustrate systems with heavy reliance on out-of-pocket (OOP) payment, with Nigeria having limited pooled financing and Singapore combining substantial cost-sharing with universal coverage and targeted subsidies.

Table 1 summarizes the four health system models using the organizing framework of who pays, who owns, who provides, and who decides. Table 2 complements this framework by comparing pharmacist authority, reimbursed services, payment mechanisms, and evidence supporting pharmacist service implementation across the selected country examples.

**Table 1.** Comparison of healthcare systems.

Dimension	Beveridge	Bismarck	National health insurance	Out-of-pocket
*Who pays? (Financing/risk pooling)*	General taxation; government finances services as single payer	Mandatory social contributions (employer/employee) into regulated insurance funds.	Government as sole payer (state-run insurance financed via taxes/contributions).	Individuals pay at point of service without pooled insurance.
*Who owns? (Ownership of production)*	Public ownership of many hospitals/clinics; pharmacies usually private.	Predominantly private ownership of hospitals, clinics, and pharmacies.	Mostly private ownership of delivery infrastructure; payment centralized.	Largely private ownership; limited organized public infrastructure.
*Who provides? (Workforce organization)*	Public employees and contracted private providers deliver care; regulated workforce.	Private providers (physicians, hospitals); services reimbursed via insurance funds.	Private providers deliver care under government reimbursement.	Independent individual providers; services bought directly by patients.
*Who decides? (Governance and coverage decisions)*	Government defines benefits and payment rules centrally.	Negotiated among insurers, providers, and regulated by law.	Government defines covered benefits and sets payment schedules.	Individual consumers make decisions; no systematic coverage rules.
Example countries	Cuba, Italy, New Zealand, Spain, Sweden, United Kingdom	Belgium, France, Germany, Japan, Switzerland	Canada, South Korea, Taiwan	Nigeria, Singapore

**Table 2.** Pharmacist roles and remuneration in example countries.

Jurisdiction	Financially supported pharmacist services	Practice authority	Funding source	Funding mechanism	Eligibility and documentation	Evidence supporting funded services
England^a^	Pharmacy First (minor illness consultations and urgent medicine supply);^2^ New Medicine Service;^3^ Hypertension Case Finding Service;^4^ Pharmacy Contraception Service;^5^ Discharge Medicines Service;^6^ seasonal vaccination service^7^	Pharmacists provide services under nationally commissioned NHS service specifications. Pharmacist independent prescribers may independently assess and prescribe within their area of competence.	NHS England through the Community Pharmacy Contractual Framework	Nationally negotiated service payments, including consultation fees, activity-based payments, fixed monthly payments tied to service delivery, and vaccine administration fees	Community pharmacies must hold an NHS contract, enroll in each service, comply with national service specifications, maintain pharmacist competency and required training, document encounters in approved electronic systems, submit claims electronically, and communicate relevant clinical information to the patient's general practitioner. Additional credentialing is required for pharmacist independent prescribing.	New Medicine Service increased proportion of patients adhering to new medicine by 10% with evidence of cost effectiveness.^8,9^ Evaluation of the hypertension case finding service found large numbers of patients were screened with 44% in low-income areas which was modeled to have prevented 600 myocardial infarctions and strokes in the first year.^10,11^ Pharmacy First reported substantial utilization, improved access to care, and reduced demand on general practitioners for management of minor illness.^12^
Germany	Comprehensive medication review for patients with polypharmacy; blood pressure management for patients receiving antihypertensives; inhaler technique assessment and training following initiation or device change; medication review and follow-up for patients receiving oral anticancer therapy; and medication review and follow-up for patients receiving immunosuppressive therapy after; influenza vaccination to adults and COVID-19 vaccination to individuals aged 12 years or older	Pharmacists independently provide pharmaceutical care services within their professional scope; diagnosis and prescribing remain physician responsibilities. Medication review services and influenza and COVID-19 vaccinations require advanced training and meeting statutory requirements.	Statutory Health Insurance funds and private insurers, as required under the Law to Strengthen Local Community Pharmacies. Approximately €150 million per year was established for most of these services. Vaccination services are funded separately by statutory health insurance under national and insurer-specific vaccination agreements.^13,14^	Pharmacies receive nationally established, fixed fee-for-service payments	Pharmacies must meet nationally defined service specification. Some services require pharmacists to complete certified advanced training. Standardized documentation and eligibility criteria apply.	National reimbursement began in June 2022. Early studies report widespread pharmacy participation.^15^ Limited data on effectiveness. Inhaler training services were effective at identifying errors and patient satisfaction was high.^16^ Medication review for polypharmacy was effective in pilot projects, reducing drug-related problems for patients.^17^
Canada^a^	Publicly funded pharmacist services vary by province but commonly include medication reviews, minor ailment assessment and prescribing, prescription adaptation/renewal, immunizations, smoking cessation, chronic disease management. Some provinces also reimburse point-of-care testing and laboratory test ordering.^18^	Most provinces authorize independent prescribing for minor ailments and prescription adaptation.	Provincial ministries of health or drug plans; selected services may also be reimbursed by private insurers	Predominantly fee-for-service.	Provincial licensure; training requirements vary; documentation in the patient record; submission of province-specific billing codes; compliance with service-specific eligibility criteria	Minor ailment prescribing improves access to care, has high patient satisfaction, reduces physician and emergency department visits.^19,20^
South Korea	National Health Insurance reimbursement is primarily limited to dispensing, medication review associated with dispensing, compounding, and medication counseling required for safe dispensing. Expanded pharmacist services are generally not reimbursed, though pilot programs have begun.^21^	Diagnosis and prescribing remain physician responsibilities and pharmacists have limited authority to independently provide reimbursed clinical services	National health insurance plus patient cost sharing; primarily dispensing fees rather than payment for clinical services	Fee-for-service reimbursement based on dispensing fees, prescription complexity, and medication management activities related to dispensing.	Pharmacies must be licensed and submit electronic claims to the Health Insurance Review and Assessment Service.	Limited evidence due to relatively few reimbursed cognitive services; interest in expanding pharmacist roles
Nigeria	Pharmacists provide clinical services, but few pharmacist clinical services are directly and routinely reimbursed.^22^	Pharmacists independently provide pharmaceutical care within their professional scope but do not have independent prescribing authority.	Most financial support is through salaries, hospital positions, public health programs, donor-funded initiatives, or direct patient payment.	Salary-based compensation or out of pocket^22^	Program-specific documentation may be required	Professional organizations advocate for recognition and remuneration for pharmacy services
Singapore	Pharmacists provide clinical services, but few pharmacist clinical services are directly and routinely reimbursed.	No independent prescriptive authority; credentialed pharmacists may prescribe as collaborative prescribers under a collaborative practice agreement that defines conditions, formulary, and escalation criteria, typically within institutions.	Ministry of Health funding through public institutions; salary-based models; limited direct fee-for-service reimbursement; direct patient payment	Salary based compensation or out of pocket	Institutional protocols; documentation in electronic medical record	Evidence demonstrates reduced hospital readmissions with pharmacist involvement, although direct reimbursement models are not established^23-25^
United States^a^	Medication therapy management; chronic disease management; immunizations; anticoagulation management; medication reconciliation; transition-of-care services; point-of-care testing; smoking cessation; services delivered under collaborative practice agreements; some states and payers also support pharmacist prescribing for specified conditions, contraception, or minor ailments	Highly variable by state. All states permit some form of collaborative medication therapy management, but the scope varies substantially. Pharmacists generally lack independent Medicare Part B provider status for most services.	Medicare; Medicaid; commercial insurers; employers; fee-for-service; value-based payment; contracts; salaries; OOP	Fragmented and payer-dependent	Requirements vary by payer and state. Provider enrollment where applicable; billing requirements; documentation; collaborative practice agreements in many states	Extensive evidence demonstrating improved clinical outcomes, cost savings, reduced hospitalizations, and high patient satisfaction, but reimbursement remains fragmented

Abbreviation: NHS, National Health Service.

^a^Examples reflect nationally available or predominant reimbursement mechanisms. Regional and provincial variation exists, particularly in Canada, the United Kingdom, and the United States.

The table summarizes nationally funded or predominant pharmacist service models. It is intended to compare financing mechanisms for pharmacist-provided clinical services rather than provide an exhaustive description of each country's pharmacy scope of practice. Where regional variation exists, the predominant national framework is presented.

## Health system models

Healthcare systems are commonly described using four stylized models: Beveridge, Bismarck, national health insurance, and OOP systems.^26-28^ While these labels summarize differences in organizing and financing, most countries combine elements of multiple models in practice.

To understand how these models function in practice, it is useful to ask a common set of foundational questions that underlie all four systems: ***who pays*** for care, ***who owns*** the delivery infrastructure, ***who provides*** services, and ***who decides*** which services are covered and how they are reimbursed.^28-30^ These questions are universal and clarify financing and governance influence how pharmacists are integrated into reimbursed roles.

### Who pays?

Across all four health system models, the question of who pays for care concerns the source of funds and the mechanism by which financial risk is pooled across individuals and populations. Beveridge systems rely on tax-financed care; Bismark systems on social insurance; national health insurance on single-payer financing; and OOP systems on direct patient payment.^31,32^

### Who owns?

Ownership refers to who controls the hospitals, clinics, and pharmacies that produce care. Beveridge systems emphasize public ownership, though private ownership often persists in community settings.^31,33^ Bismarck and national health insurance systems rely largely on privately owned, often nonprofit, infrastructure even when financing is publicly regulated.^30^ Out-of-pocket (OOP)-dominant systems are largely private and market driven. These arrangements determine whether health professionals work as public employees, contracted providers, or independent practitioners.

### Who provides?

The question of who provides care concerns both the organization of the workforce and the regulatory authority that defines scope of practice. Whether pharmacists provide clinical services such as prescribing, medication management, or preventive care depends on how professional authority is allocated and enforced. Scope of practice varies substantially across jurisdictions, even among countries with similar health system structures, so system type alone does not explain what pharmacists are permitted to do.^34^

### Who decides?

The question of who decides refers to the authority that defines covered benefits, sets payment rules, and determines which services and providers are eligible for reimbursement, often called “gatekeeping.”^28^ In Beveridge systems, these decisions are centralized in national or regional governments and prices are set administratively. Bismarck systems distribute authority across social insurance funds, provider associations, and regulators, with coverage and payment terms set through negotiation. National health insurance systems concentrate purchasing authority in a single public payer while care remains privately delivered. In OOP-dominant systems, these decisions are largely left to individuals, which limits coordination of coverage at the population level.

## Overview of the Beveridge system and implications for pharmacy practice

A Beveridge model closely aligns to the concept of universal healthcare coverage, free at the point of use (or access) to the health system and equally accessible for all citizens. The State acts as the main financier and often (but not always) the main provider, owning hospitals and clinics and organizing services centrally or through publicly accountable bodies. Some example countries that use the Beveridge model are the United Kingdom, Spain, Italy, Cuba, and New Zealand.

### England

Healthcare policy and pharmacist service commissioning are devolved across the four nations of the United Kingdom. England is presented here because it has implemented several nationally commissioned pharmacist services through the National Health Service (NHS), providing a clear example of how a Beveridge-type system can support reimbursed pharmacist care.

The Pharmacy First program in the United Kingdom represents a recent example of how a Beveridge-type health system can expand pharmacist roles through centralized policy and funding mechanisms. Introduced by the NHS in 2024, Pharmacy First enables community pharmacists to independently assess and treat patients with selected common conditions, such as uncomplicated urinary tract infections, sinusitis, sore throat, and minor skin conditions.^2^ This model illustrates how centralized decision-making and public purchasing authority within a Beveridge system can rapidly scale pharmacist services when scope of practice and reimbursement are coordinated at the national level.^35^

In addition to acute care assessment and treatment through Pharmacy First, community pharmacists in England may provide nationally funded services such as the New Medicines Service, hypertension case-finding, vaccination services, contraception services, discharge medicines support, and other services as locally needed. These services are commissioned through nationally defined specifications that establish eligibility criteria, documentation expectations, and reimbursement arrangements. Collectively, they illustrate how pharmacist services can be incorporated into routine care through centralized purchasing and standardized service design.

## Overview of the Bismarck model and implications for pharmacy practice

Bismarck systems achieve universal coverage financed primarily by employer and employee payroll contributions and administered through regulated, not-for-profit insurance funds. Government sets the statutory framework, but care is delivered largely by private providers.^27,30,36^ Pharmacists typically operate as independent providers integrated into nationally coordinated payment structures through collective contracting, with roles formalized in legislation or national agreements rather than local arrangements or time-limited pilots.^29,30^ Examples include Germany, France, Belgium, and Japan.

### Germany

Coverage and payment decisions in Germany are not made unilaterally by government but through structured negotiation among statutory insurers, provider associations, and regulators within a legally defined framework.^13,29^ Statutory health insurance covers most of the population, and community pharmacies contract collectively with sickness funds under nationally defined terms.^35^ Dispensing remuneration is tightly regulated, but pharmacists are also paid directly for specific patient care services recognized in federal law, payments that are separate from medication margins and reflect a deliberate shift toward remunerating clinical work rather than product sales.^14,37^

Since 2022, Germany has implemented a nationally standardized framework for reimbursed pharmacist services focused on medication safety, chronic disease management, and patient education.^14^ These services are reimbursed on a fee-for-service basis under statutory health insurance and are supported by clearly defined eligibility criteria, service specifications, and documentation requirements.

Reimbursed services include structured medication reviews for patients with polypharmacy, particularly those at increased risk of adverse drug events.^14^ Pharmacists are also compensated for medication counseling services for patients initiating complex therapies, including oral anticancer agents and immunosuppressive medications following organ transplantation.^14^ Additional remunerated services include inhaler technique training for patients with respiratory diseases and blood pressure monitoring and counseling for individuals with hypertension.^14^

A notable feature of this framework is its national consistency. Because reimbursement terms are established at the federal level, pharmacist services are available across regions without the need for individual payer negotiations or local commissioning arrangements.^13,14^ This consistency facilitates incorporation of clinical services into routine pharmacy practice and contrasts with systems in which pharmacist payment remains fragmented or dependent on short-term funding mechanisms.^37^ Reimbursement was also introduced alongside explicit regulatory recognition of the clinical responsibilities involved, so the scope of practice authority and payment eligibility are aligned.^14^ This alignment addresses a common policy gap observed in other systems, where pharmacists may be authorized to provide services but lack sustainable reimbursement pathways.

The German experience shows that financially supported pharmacist roles can be embedded in a multi-payer, insurance-based system without public ownership or centralized employment, provided reimbursement is negotiated into statutory benefit structures rather than treated as a discretionary add-on.^14,37^

## Overview of the national health insurance model and implications for pharmacy practice

National health insurance programs are typically organized as single-payer systems that provide uniform, comprehensive coverage to all eligible residents within a defined population. While financing and insurance coverage are centralized, the delivery of healthcare services is often decentralized and provided through a mix of public and private entities. The proportion of public vs private providers varies by country. Examples of countries that use the national health insurance model are Canada, South Korea, and Taiwan.

### Canada

In Canada, healthcare system funding is a three-layer system. Approximately 70% of total health expenditures are publicly financed, primarily through general taxation. This public funding supports universally covered, medically necessary hospital, diagnostic, and physician services. The remaining 30% of healthcare spending is privately financed, divided between OOP payments by patients and private supplemental insurance that covers services outside the core public benefit, such as outpatient prescription drugs, dental care, and vision services.^38^

### South Korea

South Korea's healthcare system is financed primarily through a mandatory national health insurance mechanism, which provides universal population health coverage and operates as a single-payer system. The healthcare system's funding is derived mainly from income-based health insurance contributions, supplemented by OOP payments made by patients at the point of care.^21^

### Pharmacy practice funding models

In Canada, community pharmacies are typically privately owned, similar to the United States, while hospital pharmacies are government operated as part of the public healthcare system. Institutional (hospital) pharmacies are reimbursed primarily through the public healthcare system, while outpatient medications are typically covered through private insurance or paid OOP by patients. Reimbursement generally includes the cost of the medication and professional dispensing fees. Patients with private insurance have prescriptions billed to their insurance, with pharmacies receiving payment in a similar manner. When no coverage exists, patients pay OOP for both the drug and dispensing fees. This system allows private pharmacies to operate as businesses while integrating with the publicly funded healthcare system for reimbursement.^39^

South Korean pharmacies are also predominantly privately owned businesses. Similarly to the Canadian system, pharmacy services are reimbursed under the national health insurance system. Covered prescription drugs are reimbursed at government-set prices, with patients typically paying a coinsurance, if required.

National health insurance systems show both the promise and the limits of centralized financing for pharmacist services. Single-payer structures simplify reimbursement and promote equitable access to core medical services, but where outpatient medications and pharmacy services fall outside the covered benefit, centralized payment alone does not support pharmacists. Sustainable financing requires that pharmacist services be named explicitly in the national coverage definition.

## Overview of the OOP model and implications for pharmacy practice

OOP models of healthcare usually occur in one of two ways: limited insurance availability or public funding in low- and middle-income countries; or high insurance deductibles and co-pays for which the patient is responsible in middle- and high-income countries. Examples of countries with high OOP include Nigeria and Singapore.

### Nigeria

Despite its largely modern capital city, Nigeria was ranked 187 of 191 countries included in the WHO health system 2000 survey.^40^ The 2022 National Health Insurance Act sets universal healthcare by 2030 as a goal; however, federal funding is far from adequate to realistically meet this goal.^41^ As a result, Nigerians spent an average of $48.05 OOP on healthcare in 2023, which accounts for 71.90% of healthcare expenditures.^42^ The average annual income for Nigeria is $1700, and 97% of Nigerians do not have health insurance.^43^ The public system operates in three tiers, but its condition leads those who can afford it to buy private plans, pay OOP at high-end facilities, or seek care abroad.^44^ The public system in Nigeria does accredit some private health facilities to provide covered services, including 66% of community pharmacies.^45^

Drug access is further limited by import dependence, supply chain failures, inflation, and counterfeits, which may make up a third of medications in circulation.^46^ Accredited pharmacies are not evenly distributed across the country with 21.4% of them concentrated in the Lagos area and five regions having none at all.^45^

### Singapore

In contrast with Nigeria, the WHO ranking of healthcare places the Republic of Singapore at sixth among 191 countries.^40^ It combines universal public insurance for large medical bills, a mandatory medical savings program for all employed persons, income-based subsidies, and a private sector for those who choose it.^47-49^ High deductibles and co-pays that increase with the increased claims are viewed as a way to decrease usage. As a result, the Commonwealth Fund 2017 estimate for annual OOP expenses for individuals in Singapore is $1273.^50^

Pharmacists in Singapore can become collaborative prescribers after completing a training program and credentialing. They practice under a collaborative practice agreement that defines the covered conditions or patient groups, formulary, permitted clinical decisions and tests, escalation criteria, and exclusions. These pharmacists are typically employed by institutions.

Nigeria and Singapore show how OOP reliance shapes pharmacist roles differently depending on the degree of public subsidy and regulation. Where insurance coverage and public financing are weak, pharmacist services are constrained by patients' ability to pay. Where cost-sharing sits within a subsidized, regulated framework and pharmacists are institutionally employed, more advanced roles are possible. In both cases, the absence of fully pooled financing limits how far pharmacist services can be standardized and routinely reimbursed.

## Applicability of each pharmacy model to US pharmacy practice

The US health system contains elements of all four models. An estimated 337 400 pharmacists practiced in the US in 2023, representing 0.26% of the workforce.^51,52^ Employer-based and other private insurance covers 54% of the population, Medicare (federal coverage for adults over 65 and some people with disabilities) 19%, and Medicaid (state-administered coverage for low-income individuals, jointly financed with federal funds) 18%, with the remainder covered by direct-purchase plans, military and veterans' programs, or uninsured and paying OOP.^53^

A key difference when comparing international pharmacy practice to the US is the significant impact of state-level regulation in the United States. Among the 50 states, governments and regulatory agencies/boards for each state have significant authority over how the practice of pharmacy is regulated. A significant barrier remains for pharmacists at the federal level; namely, their lack of recognition as healthcare providers that precludes their billing for services as healthcare providers for Medicare Part B (outpatient providers).^54^ While previous examples cite Medicare as the primary example of parallels to a US-based national health insurance, much innovation and services provided by pharmacists may be funded or supported at the state level. As a result, many models for pharmacist payment or the provision of clinical services vary based on each state's legislative and regulatory environment.^55^ As a result, it will take a combination of federal and state efforts—including examining Beveridge, Bismarck, national health insurance, and OOP models—to advance US pharmacy practice.

## Limitations and uncertainties

No country exemplifies a “pure” version of the model to which it is assigned, and most of these models took shape after World War II, when medications were inexpensive and pharmacy benefits were a small share of spending. This analysis is descriptive and intended to inform policy discussion, not to evaluate comparative effectiveness. Published evidence varies across jurisdictions, and the examples differ in maturity, scale, and evaluation methods, so the findings should not be read as evidence that any approach causes greater workforce sustainability, better outcomes, or economic efficiency.

## Recommendations: lessons from international experience and implications for US policy

Drawing on international experience across Beveridge, Bismarck, National health insurance, and OOP-dominant health systems, several observations emerge that may inform future US pharmacy policy. This comparative analysis was not intended to identify a single “best” health system. The countries examined differ substantially in their political institutions, financing arrangements, regulatory frameworks, and cultural expectations regarding healthcare. The authors recognize there are constraints on transferring policies across health systems with these differing contexts such that a system developed in one country cannot be duplicated directly to work in another country. However, several consistent lessons emerge regarding the conditions under which pharmacist services become financially supported and integrated into routine care.

### Lessons from international experience

Pharmacist services appear most durable when reimbursement is embedded in the broader financing and governance structures rather than pilot-based or through limited grants or contracts. In this way, regional variation is reduced and service delivery becomes routine. Countries that have successfully expanded reimbursed pharmacist services typically have defined reimbursement models linked to recognized professional responsibilities.

Second, scope of practice should be aligned with reimbursement. International examples show expanding scope of practice alone does not guarantee integration into healthcare delivery. Pharmacist roles are most effective when financial, policy, and regulatory models develop in tandem. This lockstep development reduces ambiguity and promotes workflow integration. The examples also suggest that the nature of pharmacist authority may influence how reimbursement mechanisms develop. In jurisdictions where pharmacists have independent authority to provide defined clinical services, direct reimbursement pathways may be easier to establish because pharmacists are recognized as accountable service providers. In contrast, collaborative or delegated practice models can support expansion of pharmacist roles while maintaining physician oversight, but reimbursement may be linked to institutional funding, physician billing arrangements, protocol-based care, or payer-specific contracts. Neither approach is inherently superior; however, the relationships between professional authority and payment mechanisms should be considered during policy design.

Third, standardized service definitions, documentation requirements, and quality expectations are present in countries that provide sustainable pharmacist services. This standardization enhances accountability, outcome evaluation, and increases payer confidence in the pharmacist services.

Finally, the international examples illustrate that health system context matters. Pharmacist payment models are products of broader environments and cannot be simply transplanted from one country to another. The value of comparative analysis lies not in identifying a model to replicate, but in understanding the underlying policy mechanisms that enable pharmacist services to be recognized, financially supported, and sustained

### Implications for US policy

One priority is the development of consistent reimbursement pathways for pharmacist-provided services. International experience suggests that durable payment models are those that move beyond limited demonstration projects and establish predictable mechanisms for ongoing reimbursement.

A second priority is strengthening alignment between pharmacist authority and payment eligibility. Expanding pharmacists' scope of practice may have limited impact if reimbursement mechanisms are not in place, are inconsistent, or are administratively burdensome. Independently provided pharmacist services and collaborative practice models may require different billing, documentation, and accountability structures, thus attention should be given to whether payment mechanisms accommodate both models.

Third, broadly adopting standardized service definitions, documentation expectations, and outcome measures could support payer confidence and facilitate implementation in diverse practice settings. This standardization would simplify administrative burden, reduce variability in reimbursement, and improve evaluation of care.

## Conclusions

The 2025 National Academies pharmacy workshop highlighted the need to address financial sustainability and workforce integration in US pharmacy. Across the international cases reviewed here, the sustainability of pharmacist services depended less on scope-of-practice expansion alone than on whether reimbursement authority, regulatory frameworks, and health system financing evolved together. No single model offers a template for the United States, but as pharmacy closures and workforce strain continue, these cases identify structural features worth considering as policymakers design payment pathways for pharmacist services.

## Supplementary Material

qxag277_Supplementary_Data

## References

[1] National Academies of Sciences, Engineering, and Medicine . Innovations in Pharmacy Training and Sustainable Practice to Advance Patient Care: Proceedings of a Workshop. The National Academies Press; 2025. 41505578

[2] NHS England . Pharmacy first. Accessed August 24, 2026. https://www.england.nhs.uk/primary-care/pharmacy/pharmacy-services/pharmacy-first/

[3] NHS England . Advanced service specification—NHS New Medicine Service (NMS). Accessed August 24,2026. https://www.england.nhs.uk/publication/advanced-service-specification-nhs-new-medicine-service-nms/

[4] NHS England . NHS community pharmacy hypertension case-finding advanced service. Accessed August 24, 2026. https://www.england.nhs.uk/publication/advanced-service-specification-nhs-community-pharmacy-hypertension-case-finding-advanced-service/

[5] NHS England . NHS pharmacy contraception service. Accessed August 24, 2026. https://www.england.nhs.uk/publication/nhs-pharmacy-contraception-service/

[6] NHS England . NHS discharge medicines service. Accessed August 24, 2026. https://www.england.nhs.uk/primary-care/pharmacy/pharmacy-services/nhs-discharge-medicines-service/

[7] NHS England . Service specification: Community pharmacy seasonal vaccination services—COVID-19 and influenza vaccination advanced services. Accessed August 24, 2026. https://www.england.nhs.uk/publication/service-specification-community-pharmacy-seasonal-vaccination-services/

[8] Elliott RA, Boyd MJ, Salema N-E, et al Supporting adherence for people starting a new medication for a long-term condition through community pharmacies: a pragmatic randomised controlled trial of the New Medicine Service. Br Med J Qual Saf. 2016;25(10):747–758. 10.1136/bmjqs-2015-004400 26647412

[9] Elliott RA, Tanajewski L, Gkountouras G, et al Cost effectiveness of support for people starting a new medication for a long-term condition through community pharmacies: an economic evaluation of the New Medicine Service (NMS) compared with normal practice. Pharmacoeconomics. 2017;35(12):1237–1255. 10.1007/s40273-017-0554-9 PMC5684280 28776320

[10] UCLpartners . Time for a step change in CVD prevention. Accessed August 25, 2026. https://uclpartners.com/time-for-a-step-change-in-cvd-prevention/

[11] Company Chemists' Association . Community pharmacies expected to deliver 15m blood pressure checks by 2026. Accessed August 25, 2026. https://thecca.org.uk/community-pharmacies-expected-to-deliver-15m-blood-pressure-checks-by-2026/

[12] Meng W, Sonnex K, Pehlivanli A, et al Use of the pharmacy first service in England in the first 12 months: geographic variation and health system context. MedRxiv 26355952. 10.64898/2026.06.18.26355952, June 22, 2026, preprint: not peer reviewed.

[13] Busse R, Blümel M. Germany: health system review. Health Syst Transit. 2014;16(2):1–296. 25115137

[14] Schulz M, Griese-Mammen N, Müller U. Clinical pharmacy services are reimbursed in Germany: challenges of real world implementation remain. Int J Clin Pharm. 2023;45(1):245–249. 10.1007/s11096-022-01492-7 PMC9938802 36394784

[15] Kroenert A-C, Bertsche T. Implementation, barriers, solving strategies and future perspectives of reimbursed community pharmacy services—a nationwide survey for community pharmacies in Germany. BMC Health Serv Res. 2024;24(1):1463. 10.1186/s12913-024-11745-y PMC11590365 39587619

[16] Kroenert A-C, Freyberg L, Sehmisch C, Michael S, Bertsche T. Community pharmacy service for patients with inhaled medications: a multi-perspective observation and assessment under routine conditions. J Eval Clin Pract. 2025;31(6):e70271. 10.1111/jep.70271 PMC12416124 40919731

[17] Seidling HM, Send AFJ, Bittmann J, et al Medication review in German community pharmacies—post-hoc analysis of documented drug-related problems and subsequent interventions in the ATHINA-project. Res Soc Adm Pharm. 2017;13(6):1127–1134. 10.1016/j.sapharm.2016.10.016 27816566

[18] Houle SKD, Grindrod KA, Chatterley T, Tsuyuki RT. Paying pharmacists for patient care: a systematic review of remunerated pharmacy clinical care services. Can Pharm J (Ott). 2014;147(4):209–232. 10.1177/1715163514536678 PMC4212445 25360148

[19] Mansell K, Bootsman N, Kuntz A, Taylor J. Evaluating pharmacist prescribing for minor ailments. Int J Pharm Pract. 2015;23(2):95–101. 10.1111/ijpp.12128 24930999

[20] Kim JJ, Tian AH, Pham L, et al Economic evaluation of pharmacists prescribing for minor ailments in Ontario, Canada: a cost-minimization analysis. Int J Pharm Pract. 2021;29(3):228–234. 10.1093/ijpp/riab006 PMC8152214 33778880

[21] Lee T-J, Hwang I, Kim H-L. Equity of health care financing in South Korea: 1990-2016. BMC Health Serv Res. 2021;21(1):1327. 10.1186/s12913-021-07308-0 PMC8665605 34895226

[22] Jackson IL, Isah A, Arikpo AO. Willingness to pay for clinical pharmacy services among people with diabetes in Nigerian community pharmacies. Value Health Reg Issues. 2023;35:95–101. 10.1016/j.vhri.2023.01.008 36933549

[23] Lee EM, Toh YF, Lim NWX, See QY. Impact of pharmacist-provided medication therapy management service on reducing unplanned readmissions in adult patients in Singapore. J Am Coll Clin Pharm. 2023;6(11):1216–1224. 10.1002/jac5.1863

[24] Chia BY, Cheen MHH, Gwee XY, et al Outcomes of pharmacist-provided medication review in collaborative care for adult Singaporeans receiving hemodialysis. Int J Clin Pharm. 2017;39(5):1031–1038. 10.1007/s11096-017-0528-1 28828593

[25] Ong KY, Cheen MHH, Chng JSG, et al Effectiveness of a multidisciplinary home-based medication review program in reducing healthcare utilization among older adult Singaporeans. Geriatr Gerontol Int. 2017;17(2):302–307. 10.1111/ggi.12706 26790376

[26] Beckfield J, Olafsdottir S, Sosnaud B. Healthcare systems in comparative perspective: classification, convergence, institutions, inequalities, and five missed turns. Annu Rev Sociol. 2013;39(1):127–146. 10.1146/annurev-soc-071312-145609 PMC5536857 28769148

[27] Wendt C, Frisina L, Rothgang H. Healthcare system types: a conceptual framework for comparison. Soc Policy Adm. 2009;43(1):70–90. 10.1111/j.1467-9515.2008.00647.x

[28] Rushton M . Who pays? Who benefits? Who decides? J Cult Econ. 2008;32(4):293–300. 10.1007/s10824-008-9083-x

[29] Frenk J, Donabedian A. State intervention in medical care: types, trends and variables. Health Policy Plan. 1987;2(1):17–31. 10.1093/heapol/2.1.17 10282125

[30] van der Zee J, Kroneman MW. Bismarck or Beveridge: a beauty contest between dinosaurs. BMC Health Serv Res. 2007;7(1):94. 10.1186/1472-6963-7-94 PMC1934356 17594476

[31] Harris B . The world has 4 key types of health service—this is how they work. World Economic Forum. Updated October 9, 2020. Accessed March 15, 2026. https://www.weforum.org/stories/2020/10/covid-19-healthcare-health-service-vaccine-health-insurance-pandemic/

[32] Shishkin S . How history shaped the health system in Russia. Lancet. 2017;390(10102):1612–1613. 10.1016/s0140-6736(17)32339-5 28980960

[33] Mesbahi Z, Piquer-Martinez C, Benrimoj SI, et al Pharmacists as independent prescribers in community pharmacy: a scoping review. Res Soc Admin Pharm. 2025;21(3):142–153. 10.1016/j.sapharm.2024.12.008 39732537

[34] Or Z, Cases C, Lisac M, Vrangbæk K, Winblad U, Bevan G. Are health problems systemic? Politics of access and choice under Beveridge and Bismarck systems. Health Econ Policy Law. 2010;5(3):269–293. 10.1017/S1744133110000034 20462471

[35] Kittipittayakorn C . Association between healthcare resources, healthcare systems, and population health in European countries. BMC Health Serv Res. 2025;25(1):720. 10.1186/s12913-024-11743-0 PMC12087073 40390026

[36] Kozuń-Cieślak G, Zdrazil P. Efficiency of healthcare systems in the European Union states—who performs better—Bismarckians or Beveridgeans? Eur Res Studies J. 2021;24(Special Issue 4):397–411. 10.35808/ersj/2725

[37] Hussain R, Babar Z-U-D. Global landscape of community pharmacy services remuneration: a narrative synthesis of the literature. J Pharm Policy Prac. 2023;16(1):118. 10.1186/s40545-023-00626-0 PMC10561514 37814349

[38] Martin D, Miller AP, Quesnel-Vallée A, Caron NR, Vissandjée B, Marchildon GP. Canada's universal health-care system: achieving its potential. Lancet. 2018;391(10131):1718–1735. 10.1016/S0140-6736(18)30181-8 PMC7138369 29483027

[39] Government of Canada . Private drug plans in Canada: expenditure report, 2018-2023. Patented Medicine Prices Review Board. Updated February 25, 2025. Accessed March 15, 2026. https://www.canada.ca/en/patented-medicine-prices-review/services/npduis/analytical-studies/private-drug-plans-2018-2023.html

[40] World Health Organization . The world health report 2000. Health Systems: Improving Performance; 2000. Accessed August 26, 2026. https://cdn.who.int/media/docs/default-source/health-financing/whr-2000.pdf?sfvrsn=95d8b803_1&download=true

[41] Yisa SS, Ogunniyi TJ, Dine RD. Strengthening primary health care in Nigeria: a means to achieve universal health coverage. BMJ Glob Health. 2025;10(10):e018486. 10.1136/bmjgh-2024-018486 PMC12570898 41151829

[42] World Bank Group . Out-of-pocket expenditure per capita (current US$)—Nigeria. Accessed March 15, 2026. https://data.worldbank.org/indicator/SH.XPD.OOPC.PC.CD?locations=NG

[43] Coker T . Pharmacy benefit management in Nigeria—Africa's pill pack. TC Health. Accessed March 15, 2026. https://www.tchealthng.com/thought-pieces/pharmacy-benefit-management-in-nigeria-africas-pill-pack

[44] Adesola RO, Opuni E, Idris I, et al Navigating Nigeria's health landscape: population growth and its health implications. Environ Health Insights. 2024;18:11786302241250211. 10.1177/11786302241250211 PMC11064746 38698838

[45] Okoro RN . Comparative analysis of private retail community pharmacies' participation in the National Health Insurance scheme of Nigeria and Ghana: implications for access to essential medicines. Ethiop J Health Sci. 2019;29(3):401–408. 10.4314/ejhs.v29i3.13 PMC6689714 31447509

[46] Olayisade A . The pharmaceutical industry in Nigeria: drug accessibility, affordability and competitiveness in the economy. *Munich Personal RePEc Archive*. 2025;125091. 10.33774/coe-2025-zn667

[47] Gaw B, Yeo T. Healthcare financing and reimbursement: a global review of major topics and trends. Accessed March 16, 2026. https://www.ibanet.org/document?id=Healthcare-Survey-2025-Singapore

[48] Kee A . Polyclinics. Updated March 12, 2025. Accessed March 16, 2026. https://www.nlb.gov.sg/main/article-detail?cmsuuid=9bed229d-f08b-4007-9078-259187205c77

[49] Earn LC . International health care system profiles: Singapore. The Commonwealth Fund. Accessed March 16, 2026. https://www.commonwealthfund.org/international-health-policy-center/countries/singapore

[50] Out-of-pocket health care spending per capita: Selected health and system statistics . The Commonwealth Fund 2020. Accessed March 16, 2026. https://www.commonwealthfund.org/out-pocket-health-care-spending-capita

[51] Census.gov. Updated October 2024. Accessed March 16, 2026. https://www.census.gov/library/stories/2024/10/american-pharmacists-month.html

[52] Mott DA, Bakken BK, Nadi S, et al Final report of the 2024 national pharmacist workforce survey. Pharmacy Workforce Center; 2024. https://www.aacp.org/article/national-pharmacist-workforce-studies

[53] Bunch LN, Ketema H. Health insurance coverage in the United States: 2024. U.S. Census Bureau, Current Population Reports, P60-288. U.S. Government Publishing Office; 2025. Accessed March 16, 2026. https://www.census.gov/library/publications/2025/demo/p60-288.html

[54] Social Security Act § 1861(s)(2), 42 USC § 1395x(s)(2). https://www.ssa.gov/OP_Home/ssact/title18/1861.htm

[55] Advancing Pharmacist Payment Parity Workgroup . State of the union: a review of state-based laws and regulations supporting pharmacist payment for clinical services. J Am Coll Clin Pharm. 2024;7(9):908–925. 10.1002/jac5.2008

